# Qualitative insights into ecobiosocial factors influencing colorectal cancer risk in Malaysia

**DOI:** 10.1080/21642850.2025.2493143

**Published:** 2025-04-16

**Authors:** Noor Azreen Masdor, Rozita Hod, Sharifah Saffinas Syed Soffian, Azmawati Mohammed Nawi

**Affiliations:** Department of Public Health Medicine, Faculty of Medicine, Universiti Kebangsaan Malaysia, Kuala Lumpur, Malaysia

**Keywords:** Colorectal cancer, qualitative, lifestyle, ecobiosocial, risk factor

## Abstract

**Introduction:**

Colorectal cancer (CRC) is a growing public health concern in Malaysia influenced by a complex interplay of ecological, biological, and social (EBS) factors. Despite its increasing incidence, limited research has explored how these factors interact to shape CRC risk in the Malaysian context, especially from the perspectives of affected individuals. This study explores Malaysians’ perceptions and experiences regarding CRC risk within the EBS framework.

**Methods:**

A qualitative case study approach involved in-depth interviews with twelve Malaysians aged 35–75 who had undergone colonoscopy at a university hospital. All interviews were recorded and transcribed. Data were collected until saturation was achieved. The transcripts were coded and analysed using ATLAS.ti software. The data were analysed using thematic analysis.

**Results:**

Findings revealed key themes related to ecological factors in the physical activity environment, which included the sub-themes of type, facilitators, barriers to physical activity, and food sources. The biological factors theme revealed that a family history of CRC influences experience and perception. The subthemes of social factors were sociocultural customs, misconceptions, food preparation methods, CRC-related foods, and food affordability.

**Conclusion:**

The findings highlighted the multifactorial nature of CRC risk. Understanding the aspects of EBS supports the development of targeted public health interventions to address modifiable CRC risk factors and promote prevention and early CRC detection in the Malaysian context.

## 1. Introduction

Colorectal cancer (CRC) is one of the most common cancers worldwide, with a significant increase in the incidence numbered 1.9 million in 2020 and is projected to increase to 3.2 million by 2040 (Morgan et al., 2023). In Malaysia, CRC is the second most common cancer among Malaysians after breast cancer. CRC predominantly affecting Chinese population, followed by Malay and Indians (Ministry of Health Malaysia, 2019). The increasing incidence of CRC is attributed to various factors, including lifestyle, genetic, and environmental factors (Cho et al., 2019; Kerr et al., 2017; Song et al., 2020; Yang et al., 2020). The adoption of Western dietary patterns has further escalated the public health problem of CRC in low – to middle-income countries (Akimoto et al., 2021; Schliemann et al., 2021). According to the latest National Health and Morbidity Survey of Malaysians, key risk factors include sedentary lifestyle, high body mass index, tobacco use and excessive alcohol consumption (Institute for Public Health, 2020).

**Figure 1.**
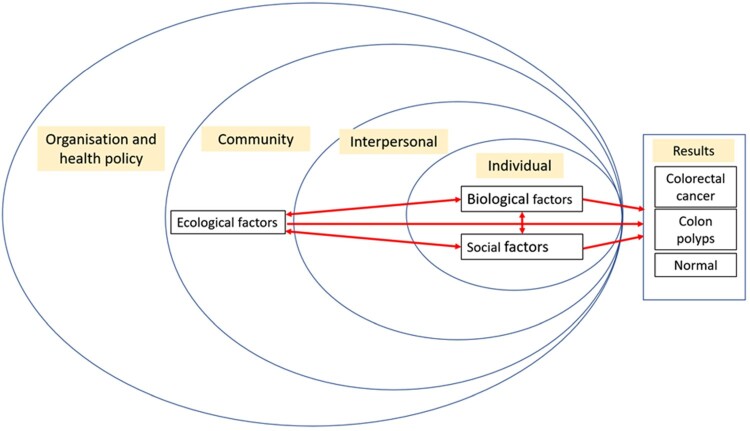
Conceptualisation of ecological, biological, and social determinants influence the formation of CRC in high-risk individuals.

The ecological factors or ‘environment’ include built and social environmental features such as population density, neighbourhood walkability, availability and accessibility of healthcare, green space and healthy food and air pollution (Canchola et al., 2017; Rodriguez-Loureiro et al., 2022). ‘Host’ or biological factors refer to the individual factors such as gender, ethnicity, age, genetic changes and family history of cancer. Social factors described as ‘agent’ that describes lifestyles and cultural practices, socioeconomic status, and health literacy, influencing behaviours like physical activities, preventive actions and dietary habits (Cho et al., 2019). Figure 1 provides an overview on how the EBS concept collectively influences the CRC in the population. The EBS framework, which incorporates ecological, biological, and social determinants of health, offers a holistic approach to understanding complicated disease processes. The framework is based on the traditional epidemiological triangle (Swinburn & Egger, 2002; Ulijaszek et al., 2016), established initially for vector-borne illnesses, and this approach is now increasingly used for non-communicable diseases such as obesity (Juarez et al., 2021). While numerous studies have examined individual risk factors, there is limited exploration of their interactions especially in multiethnic populations with diverse cultural practices, such as Malaysia.

Research on notable cultural differences among ethnic or racial groups is still lacking, particularly in certain Asian nations, even Southeast Asia (Pardamean et al. 2023). Ethnic variations in Malaysia, including Malays, Chinese, Indians, and others, may face different exposure to EBS factors necessitating nuanced investigations. Challenges arose in understanding cancer-related behaviours, as epidemiological studies often overlook these qualitative dimensions (Kerr et al., 2017). The community's perspective may highlight critical views such as the difficulty of accessing health facilities, healthier food, and physical activity, which have not been extensively explored to understand better how those risk factors are initiated and managed (Renjith et al., 2021). The qualitative study can fill this gap by uncovering how these factors are perceived, initiated and managed.

This qualitative study is part of a mixed-method study aimed at examining the influence of EBS factors on CRC risk among individuals undergoing colonoscopy in Malaysia. The quantitative component involved statistical analysis of demographic, clinical, and environmental data to identify key EBS factors associated with CRC risk. The qualitative component adopted a case study approach to explore participants’ experiences and perceptions regarding EBS influences on CRC development and progression. The integration of findings from both components offers a thorough understanding of how EBS factors interplay within the Malaysian context. A case study approach was used to explain the factors that individuals undergoing colonoscopy in Malaysia perceived as influencing CRC risk. By sharing their experiences, the study aimed to understand how the EBS factors are related to CRC and to discover specific EBS factors involved in CRC development and progression within this population. The qualitative study considered ethnic variation to explore unique EBS factors to corroborate the findings from the quantitative study. Understanding EBS factors in the Malaysian context facilitates sharing clear health messages to provide understanding and awareness of risk factors, symptoms, screening and early intervention to reduce late detection rates and control the overall increase in CRC. The study sought to fill the recent gap in exploring EBS factors, especially modifiable ones (Soffian et al., 2021). The findings aim to give evidence-based insights for establishing culturally sensitive public health interventions adapted to the Malaysian context that might be a valuable addition to research on CRC risk and support future management and prevention efforts.

## 2. Methods

A qualitative case study explored the experiences and perceptions of CRC risk among individuals categorised as non-CRC, polyps and CRC cases. This study adhered to the Consolidated Criteria for Reporting Qualitative Research (COREQ) (Tong et al., 2007) and Standards for Reporting Qualitative Research (SRQR) (O’Brien et al., 2014), which are attached as a supplementary file (S1 and S2). In-depth interviews were conducted from January to June 2022 guided by a semi-structured protocol (Timonen et al., 2018). Patients and members of the public were not involved at any step of the study's preparation, including design, administration, or conduct.

### 2.1. Patient recruitment

The participants were recruited through purposive selection from the Endoscopic Service Centre, Hospital Canselor Tuanku Muhriz (HCTM), a teaching hospital for the Faculty of Medicine, Universiti Kebangsaan Malaysia (UKM). The selection aimed to obtain diversified patients by ethnicity, gender and endoscopic findings. The participants were Malaysian, spoke either Malay or English, were 18 years old and older, and had normal colon polyps or CRC colonoscopy results. Patients with inflammatory bowel disease (IBD) and patients who did not speak Malay or English were excluded. Recruitment occurred face-to-face where team members identified eligible patients, except for two individuals who knew and had met with the interviewers, the interviewers and participants had no prior interactions with participants. Patients who chose to participate were informed about the study's objective and design, and each participant provided signed informed permission. While nine respondents were initially planned, three additional interviews were conducted to ensure data saturation, defined as the point where no new themes or subthemes emerged from subsequent interviews. Thematic analysis was conducted iteratively, with each new interview coded and compared against existing themes using Atlas.ti software. No new themes or subthemes emerged after the twelfth interview, confirming data saturation.

### 2.2. Data collection

The team members developed an interview guide based on literature reviews and expert discussions. The questions addressed EBS risk factors: environmental, social, and biological. To ensure the guide addressed the specific and culturally related aspects, the guide was based on the questions used in relevant CRC research, such as questions related to the family history of CRC based on the ‘Family History Questionnaire’ (FHQ) (Kessels et al., 2017). While CRC risk for environment and lifestyle adapted from other studies (Banna, 2018; Nawi et al., 2020; Norsa’adah et al., 2020; Power et al., 2011). The initial draft of the interview guide was developed through consultations with three experts, including public health specialists, epidemiologists and qualitative research experts. Revisions were made until the guide reached the final version. These steps were crucial as developing the right interview guide is essential to complement and align with the information gathered from the quantitative study (Table 1).

**Table 1.** Semi-structured discussion guide.

Part	Factor	Suggested time (minutes)	Questions
1	Background	2	Please explain a little about your background (personal information and diagnosis)
	Identity	2	1. What do you think a healthy life means?
2	Ecological factors and colorectal cancer	8	In your opinion, how can ecological factors influence colorectal cancer or polyps?
	Related questions		Please explain the following:
			1. How do you travel to the following places: Hospital Recreational places or fitness centres for physical activity Where to eat
			2. Tell us what motivates or hinders you while travelling.
			3. In your opinion, what influences: Hospital access Physical activity environment (green space or recreational park) Food environment (eating place)
3	Biological factors and colorectal cancer	8	In your opinion, how can biological factors influence colorectal cancer or polyps? Please describe the factors you feel are related to
	Related questions		1. Tell us about your experience with colorectal cancer or polyps that you or your family affected
4	Social factors and colorectal cancer	10	In your opinion, how can social factors influence colorectal cancer or polyps? Please describe the factors you feel are related to
	Related questions		1. In your opinion, what customs in your living culture are associated with colorectal cancer?
			2. how can cooking and preparing certain types of food contribute to colorectal cancer?
			3. What do you think about foods that can cause colorectal cancer?
			4. What kind of food can you buy at any given time?
			5. In your opinion, why don't people want to get colorectal cancer screening?
5	EBS Relevance	2	1. In your opinion, what is the relationship between ecological, biological, and social factors and colorectal cancer and polyps?

The participants were interviewed in person or via the Zoom video-conferencing platform by two team members according to the participant's preferred time and place. Two trained interviewers, N.M. (M.D.) and S.S. (M.D.), are both female medical doctors pursuing a doctoral degree in public health. The interview was conducted in Malay or English or a combination of both. At the beginning of each interview, the interviewers provided the respondents with a brief explanation and the participants’ socio-demographic backgrounds were collected. Each interview lasted approximately 20 min to an hour. Each interview was recorded audio-visually. The participants shared their responses with the interviewers, and then the interviewers shared their interpretations with the respective respondents after the interviews. Field notes were taken during the interview. The interviewers used open-ended questions to guide participants toward honest responses starting with general questions before progressing to specific and sensitive questions to minimise bias. To address potential bias, steps were taken to ensure impartiality including using a structured interview guide, maintaining professionalism, and emphasising the anonymity of responses. Reflexivity was further ensured through independent coding, peer debriefing, and team discussions, minimizing bias in data interpretation.

### 2.3. Data analysis

This study employed reflexive thematic analysis as described by Brain and Clarke to identify and interpret patterns across participant narratives, offering insights into individual and shared perspectives (Braun & Clarke, 2006, 2019). The approach facilitated flexibility and a nuanced understanding of the case study within the context of individuals undergoing colonoscopy. Data analysis followed a rigorous process to develop themes and subthemes from collected responses. First, the interviews were transcribed verbatim and imported into ATLAS.ti 21.0 software, which aids qualitative data analysis. The transcripts were read line by line to capture the essential data and obtain the initial concepts. When in doubt, the audio was played, and errors were corrected. In the event of transcription issues, the transcript was shared with the participant, who provided their opinion to ensure correct interpretation.

Coding consists of reviewing the responses and the list of quotations and assigning them the appropriate codes and categories that best fit them. The codes can be used in deductive and/or inductive analysis steps to develop themes. The researchers created a preliminary code list with known constructs and definitions. The data, i.e. the transcripts, were independently reviewed, coded, and categorised by N.M. and S.S. The findings were categorised and re-categorised using the EBS factors.

The team, which included two medical professors and two DrPH students, then discussed coding differences to create categories until a complete agreement was reached. After several rounds of coding, the research team reconvened to develop a final codebook. The team reviewed and refined the final themes to ensure they were appropriate and coherent. One team member (A.M.) examined the codes that did not fit the given themes. Then, the themes were linked to the theoretical constructs in a theoretical framework (Figure 1). The theoretical constructs were combined to form a narrative that conveyed the participants’ subjective experiences.

The essential topics were selected based on their relationship to the study question and their use in practice for manuscript writing. The participants’ quotes were selected for each theme to answer the research questions. The participants’ names were replaced with codes and numbers to ensure confidentiality (e.g. ‘C’ for CRC, ‘P’ for colon polyps, ‘N’ for normal). A professional translator translated all essential points of the Malay transcripts into English. Additionally, a team member (N.M.) compared the Malay and English versions sentence by sentence to ensure translation accuracy and to correct grammatical errors. During this last part of the writing process, sub-themes were identified when several participants described the same part of the topic with the same level of detail. The final interpretation was to link the results to the research topic. The findings were strengthened by including the team members’ evaluations and debriefings (Chametzky & Chametzky, 2016).

## 3. Results

### 3.1. Descriptive characteristic

Twelve people participated in in-depth interviews between January to June 2022. All participants were Malaysian, aged between 35 and 75, and had undergone colonoscopy. Table 2 summarises the participants’ characteristics. Most participants were Malay and female.

**Table 2.** Participants’ characteristics (*n* = 12).

No	Code	Gender	Age (in years)	Ethnicity	Diagnosis
1	RA	Female	55	Indian	Polyps
2	RB	Female	71	Chinese	Polyps
3	RC	Female	38	Malay	Normal
4	RD	Female	68	Malay	CRC
5	RE	Female	48	Malay	Polyps
6	RF	Male	78	Indian	Normal
7	RG	Female	54	Chinese	Normal
8	RH	Male	49	Malay	CRC
9	RI	Female	49	Malay	CRC
10	RJ	Male	73	Indian	CRC
11	RK	Female	67	Malay	CRC
12	RL	Male	58	Chinese	CRC

Six participants each were interviewed in person at the hospital and via Zoom. One participant refused to complete the interview due to time constraints and was excluded from the study. No re-interviews were conducted. The identified themes and sub-themes (summarised in Table 3) are described in the text, accompanied by quotes for each sub-theme.

**Table 3.** Summary of themes and subthemes from the interview.

Factors	Theme	Subthemes
Ecological	Physical activity as a protective factor	Engagement in outdoor physical activities Preference for indoor activities Irregular physical activities
	Facilitators for physical activities	Accessibility to physical activities resources Availability of facilities and its influence on participation Affordable exercise facilities and its influence on participation
	Barriers to Physical Activity	Limited access to facilities Environmental and safety concerns Fear of pain and injury
	Availability and accessibility of healthy or fast food	Outside food (Fast food restaurant and drive-thru, Mamak restaurant, Street food) Home cooked food Personal preference
Biological	Genetic risk and family history of CRC influence awareness	Awareness and preventative behaviours among family members
	Perceived risk of CRC in the family	High-risk awareness Belief in the inevitable development of CRC
Social	Cultural Belief & Custom Shaping Health Practices	Diet-related beliefs and practices Belief in traditional healers for treatment Rejection of modern treatment
	Misperception about CRC risk	Antibiotics given to poultry Lack of sleep as a contributor to CRC risk Belief on health effects of combining seafood and meat in meals
	Traditional and modern cooking methods impact CRC risk	Air fryer use health effects Harmful effects of certain cooking utensils
	Specific food associated with CRC risk	Consumption of food and association with CRC Consumption of diverse types of food (betel, spicy foods)
	Affordability Affecting Healthier Food Choices	Factor in choosing mamak restaurants (food affordable) Cost consideration in eating out/at home Food choices decision Cost-benefit of home-cooked meals
Conclusion	Interplay of EBS in CRC risk	Multiple interacting factors Diet habits perceived contribution to CRC risk

### 3.2. Ecological factors

Themes under ecological factors described how CRC risk is related to the participants’ physical activities, food accessibility and availability, and the influences that determine their choices. The participants reported their preferred type of physical activity, the environmental factors influencing the facilitators, and the barriers to physical activity. The physical activity sub-themes were classified as outdoor or indoor. The participants also addressed the physical activity facilitators and barriers. The participants mentioned accessibility regarding the sub-themes associated with food bought outside the home (outside food), home-prepared food, or suited to their personal preference for the food source environment.

#### 3.2.1. Theme 1: physical activity as a protective factor

Lower CRC risk associated physical activities. This theme highlighted how EBS factors influence their physical activity patterns and contribute to CRC risks. Participants emphasised the importance of physical activity in reducing CRC risk, yet many reported barriers that limit engagement. The participants’ physical activities were divided into outdoor and indoor activities. The participants who preferred outdoor exercise usually jogged or walked in their neighbourhood. Dumbbells were used for indoor workouts at home, such as strength training. There was little difference between the responses of the normal, polyps and CRC participants. Some participants preferred to stay indoors and had irregular physical activity for personal reasons.

Engagement in outdoor activities.

*I usually go jogging. I jog, walk, and participate in club activities near my house.*

Malay female, 68, CRC

Preference for indoor activities

*It used to be, but today it's impossible; it can only be done at home.*

Chinese female, 58, CRC

Stay indoors and exercise irregularly

*My husband is seriously ill. Since he fell ill last year, I have been worried. I stopped walking for about four or five months to care for him.*

Chinese female, 54, Normal

*I live with my mother-in-law and father-in-law. Since I am unemployed, I take care of the children. The youngest is two years old. I do homework, cook and take care of my mother-in-law. I do not do any sports.*

Malay female, 49, CRC

#### 3.2.2. Theme 2: facilitators for physical activity

Participants mentioned the features that facilitated their physical activity, such as accessibility, availability and affordability.

Accessibility and affordability of park or sports facilities.

*My son's house has a park nearby. When I go to my son's house, which lives in Subang Jaya, there are many parks and sports facilities there … .*

Indian male, 78, Normal

*I prefer a free place. I do not want to pay for the gym … *

Chinese female, 54, Normal

#### 3.2.3. Theme 3: barriers to physical activity

Participants also thought about the factors that might discourage them from being more physically active. Limited availability and environmental factors such as the weather and safety may influence their decision to be physically active.

Limited access to facilities.

*There is no recreational park near my house.*

Malay female, 67, CRC

Environment and safety concern – bad weather

*We will look at the situation. If it is the rainy season, we will not go. It is an interesting place, but make sure it is safe. I do not always go there when I see long grass. We know that the long grass contains everything.*

Malay female, 38, Normal

Fear of pain and injury

*So now I am 73 years old with a knee injury and cannot run a lot.*

Indian male, 73, Normal

#### 3.2.4. Theme 4: availability and accessibility of healthy or fast food t

This theme highlighted that the food environment has also affected CRC-related behaviours. Food availability and personal preferences influence participants’ food choices, as many frequently consume fast and outside food due to convenience and choices. Participants were asked about their food source to investigate their accessibility to food. Participants indicated that they would buy food or prepare food at home. Food prepared at home usually consisted of food that could be easily prepared, such as processed food like nuggets. Purchased food (outside the home) usually came from fast food restaurants, drive-through kiosks, street snack bars, and mamak restaurants (Indian Muslim restaurants). The typical external food items were fried, nasi lemak, fried noodles and burgers.

Outside foods.

*If outside food, it's mainly fast food; burgers, instant noodles, mamak fried noodles and so on.*

Malay female, 38, Normal

Home-cooked food

*Nuggets so quick (to prepare). Little time to cook.*

Malay female, 48, Colonic polyps

The participants stated that personal preferences regarding food ingredients influenced the choice and accessibility of certain foods.

*I do not like eating store-bought food and prefer to cook for myself. These foods contain a lot of MSG (monosodium glutamate); I do not like that.*

Malay female, 67, CRC

### 3.3. Biological factors

Biological factors related to genetic predisposition to CRC. This theme emphasises family history, personal risk perception, and preventive behaviours. The participants were asked about biological factors such as experience among family members with CRC and their perceptions concerning CRC risk. Under these factors, participants acknowledged their family history of CRC and demonstrated varying perceived risk levels.

#### 3.3.1. Theme 1: genetic risk and family history of CRC influence awareness

Two participants who have CRC reported that a family member was diagnosed with CRC and shared their personal experiences and awareness of the diagnosis of CRC as a cause of death.

Awareness among family.

*My late father had this (CRC) in 1992; he had chemotherapy, but the family did not disclose it. By the time he was diagnosed, the cancer had already spread.*

Malay male, 49, CRC

*My mother also died in this way (because of CRC). My uncle died for the same reason.*

Malay female, 68, CRC

#### 3.3.2. Theme 2: perceived risk of CRC in the family

Participants discussed the consequences of a family history of CRC, such as heritability and their belief that the cancer is not preventable. Some may have no idea of the consequences.

High risk awareness.

*I believe that CRC is inherited. If a family member has bowel cancer, I believe that the person (the descendants of the family member) will get the disease soon.*

Malay male, 49, CRC

Belief in the inevitable development of CRC

*They do not always eat fast food, but he has cancer because his family has cancer.*

Malay female, 38, Normal

### 3.4. Social factors

Under social factors, participants adhered to certain beliefs that shaped their actions toward CRC treatment and prevention. The participants reported that social factors such as existing culture and customs, misconceptions, cooking methods, risky foods, and affordable foods influenced CRC development. This theme revealed that cultural factors can interact with knowledge, action, and practices to shape CRC prevention and treatment choices.

#### 3.4.1. Theme 1: cultural belief and customs shaping health practices

Participants shared their cultural beliefs and habits about food, such as eating instant noodles, which are part of the modern lifestyle culture because they are cheaper and easier to prepare. There are many beliefs and practises, such as belief in alternative treatments, including traditional healers, and some reject modern treatments and hope for God's help.

*Eating Maggi is part of the culture because it is easy to prepare and cheap.*

Malay female, 48, Colonic polyps

Diet-related cultural beliefs and practices

*So we have wedding receptions almost every week … * *so we eat like kings and queens*.

Indian male, 78, Normal

Belief in traditional healers for treatment

*My family took me to the shaman when the illness became terrible that day; frankly, I was not ready to be taken to the shaman. The shaman confirmed (CRC) that he took me to Gerik, performed the prayer, and slaughtered the chicken. However, the shaman told me that he could not cure the disease. They just gave us back our money.*

Malay female, 68, CRC

#### 3.4.2. Theme 2: misperception of CRC risk

Participants revealed many questionable misconceptions about the belief that chickens are injected with antibiotics, that lack of sleep can lead to CRC, that dietary habits such as combining seafood with meat increase CRC risk, and that certain biscuits may contain carcinogens that lead to CRC.

*It is better (to eat meat), (la). Our chickens are given “antibiotics” to make them bigger. There are ingredients in Oreo and Ritz that can cause cancer. In my opinion, that seems to be one of the causes of cancer.*

Malay female, 38, Normal

*I have heard that one of the factors is related to the factor of sleep deprivation. Something like that.*

Malay male, 49, CRC

*Even if there is a variety of food at the event, you should choose fish or chicken (not mixed it).*

Malay female, 68, CRC

#### 3.4.3. Theme 3: traditional and modern cooking methods impact CRC risk

Participants believed that CRC was related to how food is prepared and cooked, such as using a hot air fryer with or without reference to CRC. The use of oil in cooking and pans or cooking utensils used over a long time may be associated with CRC.

Air fryer usage health effects.

*I am not sure. The air fryer seems safe because it does not use oil.*

Malay male, 49, CRC

Harmful effects of certain Cooking utensils

*We do not know (the condition of the pan), good or bad. They have used it for a long time. I assume it has something to do with the pots and pans they used, right?*

Malay female, 48, Colonic polyps

#### 3.4.4. Theme 4: specific foods associetd with CRC risk

The participants mentioned consumption of a variety of foods, including meat, fast food, and processed foods, concerning CRC.

*I also eat pork. I heard from friends I met in MLM (Multilevel Marketing) that they said, oh, do not eat so much meat; it will give you cancer. But wait. I do not think I believe that.*

Chinese female, 54, Normal

*Datuk Naidhu (a surgeon) once said that fast foods are bad for you because they contain preservatives.*

Indian female, 55, CRC

#### 3.4.5. Theme 5: food affordability affecting healthier food choices

Participants were asked to describe the type of food they could prepare or buy to explore its affordability. Participants consistently responded that they would buy from outside or prepare at home depending on their preferences, such as taste, time and type. Food from outside, e.g. from a mamak restaurant, can be cheap but should be treated cautiously.

Factors in choosing Mamak restaurant.

*The (mamak) food is delicious, cheap, and plentiful. So, you will have to buy it either way. It is just that I think I should trim it down a little (buying outside), especially the McD. We will buy from the mamak shop because it is cheap.*

Malay female, 38, Normal

*Raw food is unaffordable today. So I prefer cheap, simple, affordable, and easily accessible; it is easy to drive through.*

Malay female, 48, polyps

*My children always like what I cook. Because I think if I cook for my child, they can eat it repeatedly. Two packs of Bihun. Say RM 1.90. Then we add green vegetables, eggs and soy sauce from that money.*

Malay female, 49, CRC

### 3.5. Conclusion on the link between EBS factors and CRC

Finally, we solicited feedback on the relationship between the EBS factors and CRC, where the participants stated that the factors could be multifactorial. Some participants indicated that food was significant in CRC development.

#### 3.5.1. Multiple interacting factors for CRC risk

*If you do not sleep enough or rest enough, you will get easily irritable, overeat, or drink alcohol at night … * *All of these will affect your life (getting cancer). As far as I can tell, the lifestyle significantly impacts what you are.*

Chinese female, 54, Normal

#### 3.5.2. Diet habits perceived contribution to CRC risk

*It is related. Nevertheless, you cannot blame it all on food. Because we have no idea which one may cause it. When a man is too lazy to exercise, his body becomes bigger and heavier, he eats fast food and neglects his diet. He has no interest in learning to cook, and he has no interest in choosing (healthy) food. He has no restrictions and does not restrict his diet; he enjoys eating meat. There is indeed a connection. If someone in the family has cancer, they may get bowel cancer.*

Malay female, 38, Normal

## 4. Discussion

This study aimed to understand the influence of EBS factors on CRC risks among individuals undergoing colonoscopy in Malaysia. The findings revealed several themes and sub-themes that associated EBS factors with CRC risk, indicating their critical role in CRC initiation and development. During the interviews, the participants presented their thoughts on how the individual interacts with the environment to shape the biological and social factors influencing their behaviour and actions related to CRC.

Our findings highlight the important role of environmental factors in influencing CRC risk, mainly through their impact on physical activity and food accessibility. Participants identified key facilitators, such as the availability of parks and sports facilities, which encourage outdoor physical activities known to reduce CRC risk (Liu et al., 2016). Conversely, barriers like limited availability of recreational spaces and adverse weather conditions were noted to hinder regular physical exercise. The participants indicated that the accessibility and availability of facilities determined the type of physical activity. The accessibility and availability of a supportive environment for physical activities aided outdoor physical activities. Physical activities typically occur in specific locations such as parks, sports fields, or hiking trails, collectively termed ‘environments for physical activities’ (Sallis, 2009). Activities that require moving from one location to another decrease the likelihood of individuals engaging in physical activity, particularly in people with disabilities. In addition to the environment motivating physical activity, factors such as personal choice and behaviour also determine the individual's actions. However, People without access to a sports facility can still exercise indoors, as stated by one of the respondents. Indoor exercises, including treadmill running, dancing, and cycling, are beneficial to health, although the benefits of CRC prevention remain limited. These insights featured the importance of designing urban environments that promote accessible and safe opportunities for physical activity to mitigate CRC risk. Therefore, more research is needed on the type of physical activity and interaction that can guide policymakers in creating an environment that promotes and supports aspects (Pontin et al., 2022).

The participants emphasised the importance of the food source environment, including food accessibility. Additionally, Turner et al. established that food safety, affordability, and sufficiency are essential to good health (2018). Food accessibility means that individuals have access to adequate and nutritious food. Food choices and constraints depend on the person's socio-demographic characteristics (Wolf et al., 2021). For example, people who work outdoors are more likely to buy food and choose high-sugar and high-fat processed foods at fast food outlets and convenience stores (Congdon, 2019). Diet influences not only cancer risk but also influences CRC outcomes. Fong et al. examined the lack of access to healthy food and identified lower survival rates for CRC (Fong et al., 2021). Therefore, the critical CRC prevention factors are the physical environment of cities, such as urban design and sprawl, access to healthy food, and physical activity.

Biological factors emerged as a prominent theme, with participants frequently referencing familial or inherited predispositions to CRC. The study of biological factors depends on information such as gender, age, ethnicity, history of adenomatous polyps, IBD, and family history (Hossain et al., 2022). Respondents discussed their personal family members’ CRC experience. Some participants recognised that CRC is related to family history and that they have a higher risk. One participant worried that even if she cared for her health and maintained a healthy lifestyle, she believed she would develop CRC because of their family history. Thus, biological factors influenced participants’ awareness and understanding of CRC. Awareness of risk variables, including modifiable and non-modifiable, can lead to proactive decision-making and action, encourage adherence to recommended screening, and reduce risk with appropriate management for modifiable risk factors (Zlot et al., 2012). DNA mutations that are affected due to inherited or acquired genes increase intestinal cell growth. Genetic mutations, whether inherited or acquired, are crucial in understanding the pathogenesis of CRC, as these mutations can significantly influence intestinal cell growth and tumour development. Molecular characterisation of these mutations provides insights into disease prognosis and forms personalised therapeutic strategies, emphasising the need for genetic screening and counselling in high-risk populations (De Rosa et al., 2016).

Cultural beliefs and social customs were identified as significant determinants impacting CRC risk. Misperceptions about CRC, such as beliefs in the specific foods related to CRC or reliance on traditional healers over modern medical treatments, can adversely affect health outcomes. Cultural factors can shape dietary patterns, food quality, and nutritional intake, influencing CRC risk profile(Playdon et al., 2023). Addressing these cultural misperceptions through targeted health education could play a vital role in reducing CRC incidence. Many participants were familiar with red meat, fast food, and processed meals, which were substantially related to the risk of CRC. Goldman et al. (2009) mentioned that Hispanics similarly categorised certain foods as ‘bad’ because of their eating habits, containing high-fat foods, hot spices, alcohol, high-salt and low-fiber diets. In Malaysia, instant noodles are famous because they are affordable, easy to prepare, and fulfilling. Ramen and ramyeon noodles, like those found in other Asian countries, particularly Japan and Korea, are culturally significant gastronomic treats. However, frequent consumption of instant noodles is considered unhealthy since they contain high quantities of sodium and carbohydrates but relatively few vegetables (Kwak et al., 2021). Such food typically contains additives that may alter gut microbiota, promote inflammation, and contribute to colon carcinogenesis (Wang et al., 2022).

This research discovered several cultural beliefs, including a preference for specific food, belief in superstitious powers and shamans, and rejection of modern medicines. Kim and Lim found that other relevant cultural attitudes in the Singaporean community, such as optimism, pessimism, and superstition, were connected with cancer fatalism (Kim & Lwin, 2021), which is the belief that everything is predetermined and has been identified, which is an obstacle to health-conscious behaviour (Perfetti, 2018). However, even if a person believes their life is predetermined from birth, they can still act as necessary or try to change their ‘destiny’, as explored in a qualitative study (Lee & Lee, 2013). In the traditional setting, shamans are still considered important in certain regions in Malaysia. Despite many people knowing that shamans cannot cure cancer, some Malaysians still believe in them and engage in their services. A qualitative study by Mohd Mokhtar et al. reported that belief persisted in traditional healers or shamans, especially among the Indigenous Orang Asli (2021). It was determined that the Orang Asli held their ancestors’ beliefs, and some continued to practice animistic rituals and wisdom. In some cases, they believed that shamans could cure any disease. Further studies are needed to understand better how perceptions and fatalism related to CRC prevention behaviours are developed, especially in Asian populations.

Socioeconomic factors influence food affordability and determine food quality, choice, type, source, and purpose. Affordability can limit healthy food choices, as cheap food typically has an unbalanced ratio of macro – and micronutrients, leading to malnutrition and obesity. The quick service, affordable food choices, and cosy environment of Mamak restaurants are advantages that outweigh the drawbacks, while many others felt that outside food typically came at an additional cost (Hashim et al., 2023). Preparing food at home can also be so time-consuming that it is more convenient to buy outside food. Programmed nutrition plans for home-prepared meals can aid the low-income population in Malaysia in following a healthy, balanced diet with minimal cost according to their individual cancer care needs (Alaini et al., 2019). Promoting a healthy, balanced, affordable, and palatable diet can improve overall health and reduce cancer risk.

The three factors (environmental, biological, and social) are all involved in explaining CRC risk. Generally, the participants agreed on the relationship between the EBS factors and CRC. The factors can be multifactorial, but diet, nutrition, and specific foods might significantly influence CRC risk due to the daily exposure and food environment determined by other factors such as personal and socioeconomic backgrounds (Aran et al., 2016). This information is beneficial for better understanding the mechanisms and relationships between all other factors in CRC.

### 4.1. Strengths and limitations

This study was limited by the exclusive use of Malay and English after considering the need for translators, time, and cost. Nevertheless, the possibility of investigating a more comprehensive perspective remains if non-Malay and non-English speakers are included. A future, more extensive, multi-centre study could explore other factors associated with CRC. The study is subject to the researcher's interpretation, and the results are based on this interpretation. The restricted number of samples suggested that the information obtained could not describe the general population.

Twelve people participated in this study. The number of participants was appropriate, as Guest et al. determined from 60 in-depth interviews with workers in West Africa that approximately 70% and more than 90% of all identified themes emerged in the first six interviews and first 12 interviews, respectively (Guest et al., 2006). The present study developed themes and sub-themes among EBS factors that can aid primary prevention in strengthening health policies to provide better advice on CRC risk reduction. The study of community involvement in CRC risk suggested that tailored interventions are needed.

### 4.2. Study implications and recommendations

This qualitative study enriches our understanding of how ecological, biological, and social (EBS) factors influence CRC risk in Malaysia. By capturing participants’ perceptions and experiences, it identifies both barrier and facilitators such as cultural beliefs, food preferences and environmental limitations. The themes on a person's lifestyle choices, including physical activity and dietary practices, highlight the importance of modifiable behaviours in CRC prevention. These understandings are crucial for developing targeted health promotion strategies. Furthermore, the findings provide supportive evidence for informing current policies to address areas like access to recreational spaces, healthy food options, and culturally sensitive health education. By highlighting structural and behavioural determinants of CRC risk, this study provides policymakers with valuable information to enhance prevention and intervention strategies.

This study recommends the development of culturally appropriate education program to address CRC risk factors, emphasizing the importance of early screening and dispelling misconceptions about CRC risk (e.g. the belief that combining seafood and meat or using certain cooking utensils increases cancer risk). Additionally, revised policies that integrate EBS factors can greatly strengthen national cancer prevention efforts. For example, promoting equitable access to healthy foods and healthcare facilities while incorporating green spaces and pedestrian-friendly areas to encourage physical activity. Community-based interventions tailored to Malaysia's cultural and socioeconomic diversity are essential. Initiatives such as cooking workshops or local market programs promoting healthy food preparation should be expanded for community benefit. Targeted awareness campaigns and dialogue with specific populations can effectively address CRC relate misconceptions fostering informed health behaviours.

## 5. Conclusion

The results demonstrated the role of EBS factors in clarifying CRC risk. Our findings provided insights into understanding CRC using the EBS approach. Understanding EBS factors among Malaysians helps develop comprehensive public health interventions, and further research could provide recommendations for modifiable risk factors and the promotion of healthy behaviours, especially among Asian populations. At the same time, interventions to improve disease control should be implemented in all eligible multiethnic populations as part of a comprehensive CRC control and management strategy.

## Supplementary Material

FS2 Table EBS_SRQR_Checklist.docx

FS1_Table COREQ checklist.docx

## Data Availability

The data supporting this study's findings are available on request from the corresponding author, A.M.
