# Supplementary material for: Qualitative insights into ecobiosocial factors influencing colorectal cancer risk in Malaysia
Source: Health Psychol Behav Med. 2025 Apr 16;13(1):2493143. doi: 10.1080/21642850.2025.2493143 (PMC12006936; doi:10.1080/21642850.2025.2493143)
Supplement: FS1_Table COREQ checklist.docx [file RHPB_A_2493143_SM0655.docx]

**S1 Table: COREQ (COnsolidated criteria for REporting Qualitative research) Checklist.**

Manuscript title: Qualitative Insights into Ecobiosocial Factors Influencing Colorectal Cancer in Malaysia

| **Topic** | **Item No.** | **Guide Questions/Description** | **Reported on Page No.** |
| --- | --- | --- | --- |
| **Domain 1: Research team and reflexivity** | | | |
| *Personal characteristics* | | | |
| Interviewer/facilitator | 1 | Which author/s conducted the interview or focus group? | Page 3 L103 |
| Credentials | 2 | What were the researcher’s credentials? E.g. PhD, MD | Page 3 L103 |
| Occupation | 3 | What was their occupation at the time of the study? | Page 3 L103 |
| Gender | 4 | Was the researcher male or female? | Page 3 L103 |
| Experience and training | 5 | What experience or training did the researcher have? | Page 3 L104 |
| *Relationship with participants* | | | |
| Relationship established | 6 | Was a relationship established prior to study commencement? | Page 3 L94-95 |
| Participant knowledge of the interviewer | 7 | What did the participants know about the researcher? e.g., personal goals, reasons for doing the research | Page 3 L91,94 |
| Interviewer characteristics | 8 | What characteristics were reported about the inter viewer/facilitator? e.g., Bias, assumptions, reasons and interests in the research topic | Page 3 L113 |
| **Domain 2: Study design** | | | |
| *Theoreticalframework* | | | |
| Methodological orientation and Theory | 9 | What methodological orientation was stated to underpin the study? e.g.  grounded theory, discourse analysis, ethnography, phenomenology, content analysis | Page 2 L58 |
| *Participant selection* |  |  |  |
| Sampling | 10 | How were participants selected? e.g., purposive, convenience, consecutive, snowball | Page 3 L83 |
| Method of approach | 11 | How were participants approached? e.g., face-to-face, telephone, mail, email | Page 3 L93 |
| Sample size | 12 | How many participants were in the study? | Page 4 L149 |
| Non-participation | 13 | How many people refused to participate or dropped out? Reasons? | Page 4 L156 |
| *Setting* |  |  |  |
| Setting of data collection | 14 | Where was the data collected? e.g., home, clinic, workplace | Page 4 L152 |
| Presence of nonparticipants | 15 | Was anyone else present besides the participants and researchers? | Page 4 L154 |
| Description of sample | 16 | What are the important characteristics of the sample? e.g., demographic data, date | Page 4 Line 149-157 |
| *Data collection* |  |  |  |
| Interview guide | 17 | Were questions, prompts, guides provided by the authors? Was it pilot tested? | Page 3 L99,  L107 |
| Repeat interviews | 18 | Were repeat inter views carried out? If yes, how many? | Page 4 L156 |
| Audio/visual recording | 19 | Did the research use audio or visual recording to collect the data? | Page 3 L111 |
| Field notes | 20 | Were field notes made during and/or after the interview or focus group? | Page 3 L113 |
| Duration | 21 | What was the duration of the inter views or focus group? | Page 3 L110 |
| Data saturation | 22 | Was data saturation discussed? | Page 3 L90  Page 16 L530-536 |
| Transcripts returned | 23 | Were transcripts returned to participants for comment and/or correction? | Page 3 L111-113 |
| **Domain 3: analysis and findings** | | | |
| *Data analysis* |  |  |  |
| Number of data coders | 24 | How many data coders coded the data? | Page 4 L127-128 |
| Description of the coding tree | 25 | Did authors provide a description of the coding tree? | No coding tree |
| Derivation of themes | 26 | Were themes identified in advance or derived from the data? | Page 4 L124-128 |
| Software | 27 | What software, if applicable, was used to manage the data? | Page 3 L120 |
| Participant checking | 28 | Did participants provide feedback on the findings? | Page 3 L111 |
| *Reporting* |  |  |  |
| Quotations presented | 29 | Were participant quotations presented to illustrate the themes/findings?  Was each quotation identified? e.g., participant number | Yes, page 5-13 |
| Data and findings consistent | 30 | Was there consistency between the data presented and the findings? | Yes, for example Page 12 L382 |
| Clarity of major themes | 31 | Were major themes clearly presented in the findings? | Yes,Summary provided on Table 3 |
| Clarity of minor themes | 32 | Is there a description of diverse cases or discussion of minor themes? | Yes, Summary provided on Table 3 |

Developed from: Tong A, Sainsbury P, Craig J. Consolidated criteria for reporting qualitative research (COREQ): a 32-item checklist for interviews and focus groups. *International Journal for Quality in Health Care*. 2007. Volume 19, Number 6: pp. 349 – 357
